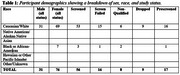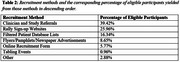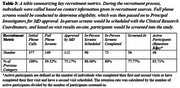# Carrying out a longitudinal multimodal biomarker study of late life depression and Alzheimer's disease risk: outreach and recruitment strategies

**DOI:** 10.1002/alz70857_105838

**Published:** 2025-12-25

**Authors:** Mayuri L. Venkatesh, Natalia Tarr, Alexa Coussoule, Natalie R Scher, Claudia R. Aibel, Onyinye Udeogu, Jacqueline Beltran, Scott R. Mackin, Gad A. Marshall, Catherine E. Munro, Jennifer R. Gatchel

**Affiliations:** ^1^ Department of Neurology, Massachusetts General Hospital/Brigham & Women's Hospital, Boston, MA, USA; ^2^ Massachusetts General Hospital/Brigham & Women's Hospital, Boston, MA, USA; ^3^ University of California, San Francisco Department of Psychiatry, San Francisco, CA, USA; ^4^ Gerontology Research Unit, Department of Geriatric Psychiatry, Massachusetts General Hospital/Harvard Medical School, Charlestown, MA, USA; ^5^ Department of Psychiatry, Massachusetts General Hospital, Harvard Medical School, Boston, MA, USA; ^6^ McLean Hospital, Harvard Medical School, Belmont, MA, USA

## Abstract

**Background:**

Understanding the relationship between depression and dementia—two leading causes of morbidity and disability—is crucial. However, clinical research in this domain faces challenges related to recruitment and outreach, stigma, risk disclosure, and diversity. *The Mood and Memory in Aging Study (MOMENT)* at Mass General Brigham (MGB), a 5‐year multimodal biomarker observational study of late‐life depression and dementia risk, has employed various strategies to enhance recruitment and outreach. We summarize these approaches and discuss future directions for optimizing research in this field.

**Method:**

Recruitment occurred in two phases: an initial K23‐funded phase, and a second R01‐funded phase that aimed to add 50 participants. Non‐demented older adults with major or persistent depressive disorder and moderate to severe depressive symptoms were recruited. Strategies included digital platforms (participant databases, website sign‐ups), clinician referrals, community outreach via social media, and partnerships with neurology clinical research cores. Participant demographics and engagement metrics from the second recruitment phase were analyzed to assess strategies and effectiveness.

**Result:**

The second recruitment phase, initially projected to finish December 2024, concluded one month early due to enhanced efforts in 2024. Expanded methods—tables at clinics and community fairs, a clinical research database of older adults interested in aging and dementia research, recruitment websites, Craigslist/newspaper ads, and referrals from MGB clinics and the Massachusetts Alzheimer's Disease Research Center—together boosted weekly call volume by 36% relative to 2023. Effective methods included clinician referrals (39.42%), recruitment websites (25.96%), clinical research databases (16.34%), ads (8.65%), websites (5.77%), tabling (0.96%). 56 participants were enrolled, with a 22.2% screen‐fail rate and a ∼85% retention. Study time commitment, travel, and reluctance to complete neuroimaging were among barriers to recruitment and retention.

**Conclusion:**

We employed a multi‐faceted strategy integrating clinician referrals, advertisements, and partnership with registries to efficiently meet recruitment milestones. Challenges including limited enrollment of Hispanic/Latino or severely symptomatic individuals, and attitudes towards neuroimaging highlight the need to optimize methods to address health disparities. Future efforts including translation of study documents, expanded database queries, and options for non‐imaging participation will improve outcomes and enhance understanding of depression and AD risk.